# Caspase-1 as a Central Regulator of High Fat Diet-Induced Non-Alcoholic Steatohepatitis

**DOI:** 10.1371/journal.pone.0056100

**Published:** 2013-02-07

**Authors:** Laura J. Dixon, Chris A. Flask, Bettina G. Papouchado, Ariel E. Feldstein, Laura E. Nagy

**Affiliations:** 1 Department of Molecular Medicine, Case Western Reserve University, Cleveland, Ohio, United States of America; 2 Departments of Radiology and Biomedical Engineering, Case Western Reserve University, Cleveland, Ohio, United States of America; 3 Department of Pathology, University of California San Diego, La Jolla, California, United States of America; 4 Department of Pediatrics, University of California San Diego, La Jolla, California, United States of America; 5 Departments of Pathobiology and Gastroenterology, Cleveland Clinic, Cleveland, Ohio, United States of America; CIMA. University of Navarra, Spain

## Abstract

Nonalcoholic steatohepatitis (NASH) is associated with caspase activation. However, a role for pro-inflammatory caspases or inflammasomes has not been explored in diet-induced liver injury. Our aims were to examine the role of caspase-1 in high fat-induced NASH. C57BL/6 wild-type and caspase 1-knockout (*Casp1^-/-^*) mice were placed on a 12-week high fat diet. Wild-type mice on the high fat diet increased hepatic expression of pro-caspase-1 and IL-1β. Both wild-type and *Casp1^-/-^* mice on the high fat diet gained more weight than mice on a control diet. Hepatic steatosis and TG levels were increased in wild-type mice on high fat diet, but were attenuated in the absence of caspase-1. Plasma cholesterol and free fatty acids were elevated in wild-type, but not *Casp1^-/-^* mice, on high fat diet. ALT levels were elevated in both wild-type and *Casp1^-/-^* mice on high fat diet compared to control. Hepatic mRNA expression for genes associated with lipogenesis was lower in *Casp1^-/-^* mice on high fat diet compared to wild-type mice on high fat diet, while genes associated with fatty acid oxidation were not affected by diet or genotype. Hepatic Tnfα and Mcp-1 mRNA expression was increased in wild-type mice on high fat diet, but not in *Casp1^-/-^* mice on high fat diet. αSMA positive cells, Sirius red staining, and Col1α1 mRNA were increased in wild-type mice on high fat diet compared to control. Deficiency of caspase-1 prevented those increases. In summary, the absence of caspase-1 ameliorates the injurious effects of high fat diet-induced obesity on the liver. Specifically, mice deficient in caspase-1 are protected from high fat-induced hepatic steatosis, inflammation and early fibrogenesis. These data point to the inflammasome as an important therapeutic target for NASH.

## Introduction

Consumption of high-energy diets and weight gain are associated with development of several metabolic complications such as insulin resistance and hepatic steatosis, characteristics of an early stage within the spectrum of Non-Alcoholic Fatty Liver Disease (NALFD). NAFLD is a common form of chronic liver disease, affecting both adults and children, which is strongly associated with obesity and insulin resistance [1], [2]. 10−20% of adults have hepatic steatosis, which is characterized by triglyceride accumulation in liver cells and follows a benign non-progressive clinical course [3], [4]. Nonalcoholic steatohepatitis (NASH) is defined as lipid accumulation with evidence of cellular damage, inflammation and early to moderate fibrosis [5]. NASH is a serious condition, as approximately 3−15% of these patients progress to cirrhosis, with complications including portal hypertension, liver failure and hepatocellular carcinoma [6], [7], [8]. In particular, the mechanisms responsible for liver injury and disease progression remain incompletely understood, but are of significant biomedical importance, as identification of cellular and molecular processes of NASH pathogenesis may help to identify novel diagnostic and therapeutic targets for this highly prevalent and potentially serious disease.

Until recently, progression from NAFL to NASH was modeled as a two-hit process. The first hit is proposed to be the accumulation of lipids within hepatocytes, and considered a requirement for later events leading to liver injury [9], [10]. Lipid accumulation occurs as a result of availability of excess free fatty acids (FA) coming from high-energy/high fat diets or released from adipose tissue, as well as an increase in hepatic fatty acid synthesis and decrease in degradation [11]. Impairment of triglyceride secretion by VLDL can also contribute to hepatic steatosis in some conditions [12]. The second hit is proposed to be a multifactorial process likely involving a combination of apoptosis and necrosis, oxidative stress and lipid peroxidation, proinflammatory cytokine and chemokine production, dysregulated adipokine expression and mitochondrial dysfunction [13]. All of these insults cumulatively contribute to the progression of NAFLD to NASH and fibrosis. Despite this characterization of multiple contributors to disease progression, there remains a dearth of evidence to explain the molecular mechanisms of how these proposed "second hits" in diet-induced obesity result in the progression from steatosis to NASH.

The inflammasome is an innate immune response that involves the formation of a multiprotein caspase-1 -activating complex [14]. The inflammasome complex contains cysteine-aspartate protease-1 (caspase-1), Apoptosis-associated speck-like protein containing a CARD (ASC), and a Nod-like receptor (NOD) protein (NLRP). The inflammasome senses danger signals through intracellular NLRPs. Among known inflammasomes (NLRP1, NLRP3, NLRC4, AIM, and IPAF), NLRP3 is responsible for the activation of immune cells in response to tissue injury and cell death via monosodium urate [15]. Formation of the inflammasome complex leads to the activation of caspase-1, which in turn proteolytically cleaves interleukin-1 (IL-1) and -18 into their mature active forms [16].

Multiple lines of evidence suggest a role for the inflammasome in NASH, for example, IL-1 receptor (IL-1R) is thought to play a role in NASH since IL-1 knockout and IL-1R knockout mice are protected from methionine and choline deficient (MCD) diet-induced steatohepatitis [17], [18], [19], [20]. We and others have shown an increase in expression and activation of caspase-1 in the MCD mouse model of NASH [17], [18], [21]. In addition, pharmacological inhibition of all caspases blocks hepatocyte apoptosis and the progression of MCD-induced fibrosis in mice, but does not improve hepatocellular injury assessed by plasma alanine aminotransferase (ALT), nor hepatocellular ballooning, steatosis or inflammation [22], [23]). While pointing to caspases as important mediators of molecular mechanisms in NASH, these data were limited to the MCD diet, which can be considered a non-pathogenic NASH model. The MCD diet induces the phenotypic changes of NASH in the liver, but does not reflect the dietary etiology, pathogenesis, or disease mechanisms seen in NASH patients [24], [25]. In addition, the MCD diet causes non-characteristic features of NASH, such as weight loss and wasting syndrome [26].

High energy/high fat diets and obesity lead to a chronic state of low-grade inflammation, hyperinsulinemia, hyperglycemia and increased ROS production and cell death. We tested the hypothesis that caspase-1 plays a role in the pathogenesis of high fat diet-induced liver injury. In order to test this hypothesis, we exposed caspase-1 deficient mice (*Casp1^-/-^*) to a high fat diet for 12 weeks. The absence of caspase-1 decreased hepatic steatosis. *Casp1^-/-^* mice were protected from high fat diet-induced hepatic inflammatory cytokine expression. In addition, absence of caspase-1 protected mice from early stages of fibrogenesis, suggesting that caspase-1 is necessary for an initial pro-fibrotic response during obesity-induced liver injury.

## Materials and Methods

### Animal Studies

These experimental protocols were approved by the Institutional Animal Care and Use Committee at the Cleveland Clinic. Male C57BL/6 mice, 20 to 25 g of body weight, were purchased from Jackson Laboratory, Bar Harbor, ME. Caspase-1, Caspase-11 double knockout mice (*Casp1* ^-/-^) on a C57BL/6 background (generated by Dr. Richard Flavell from Yale University, New Haven, CT) were described previously [27], [28]. Animals were housed in pairs in standard microisolator cages and maintained on a 12 h∶12 h light/dark cycle. Mice were placed on a high fat diet (HFAT) containing 42% kcal from fat, 18.8 kJ/g (TD 88137 Teklad Mills, Madison, WI) or a standard control diet (CTL) containing 6% fat, 13.0 kJ/g (TD 2918, Teklad Mills, Madison, WI). Body weight and food was measured at 0, 3, 6, 9, and 12 weeks. Animals in each group were euthanized after 12 weeks on respective diets. Euthanasia was performed with a 32.6% ketamine, 10.9% acepromazine and 6.5% xyalazine cocktail. Animals on CTL diet received 0.12 mL cocktail/25 g body weight. Animals on HFAT diet received 0.11 mL cocktail/25 g body weight.

### Immunoblot and Enzyme-linked immunosorbent Analysis

Frozen liver was homogenized in RIPA lysis buffer (10 ml/g tissue) containing protease and phosphatase inhibitors (sodium pyrophosphate (0.45 mg/ml), bacitracin (1 mg/ml), leupeptin (0.01 mg/ml), aprotinin (17.5 µg/ml), bestatin (5 µl/ml), vanadate (0.18 mg/ml)) and protease inhibitor cocktail (#11873580001, Roche, Indianapolis, IN). Protein concentration was determined using the BCA Protein Assay (Bio-Rad Laboratories, Hercules, CA). Lysates (∼30 µg protein) were loaded onto 6−12% reducing SDS-polyacrylamide gels and probed for caspase-1 (Millipore, Billerica, MA); IL-1β (Abcam, Cambridge, MA); P-ACC and ACC (Cell Signaling Technologies, Danvers, MA); HSC70 was used as the loading control (Santa Cruz Biotechnology, Santa Cruz, CA). Immunoreactive proteins were visualized using enhanced chemiluminescence, images collected, and signal intensities quantified using Eastman Kodak Co. Image Station 4000R. Lysates were probed for IL-1β with IL-1β ELISA Ready-SET-Go (eBioscience, San Diego, CA). IL-1β was normalized to lysate protein concentration.

### Imaging of Animals for Body Fat Analysis

Each animal was initially anesthetized with 2−3% isoflurane in oxygen. The animals were then placed in a prone position within a Bruker Biospec 7T MRI scanner (Bruker Biospin, Billerica, MA). A 72-mm diameter volume coil was used for excitation and signal detection to maximize the uniformity of the images. After localizer scans, a Relaxation Compensated Fat Fraction (RCFF) MRI acquisition and reconstruction process was used to generate quantitative fat fraction maps for each imaging slice [29]. Briefly, three asymmetric echo spin echo MRI acquisitions were acquired (TR/TE = 1500 ms/20 ms, 17–25 coronal slices, resolution = 200 um×200 um×1000 um, 2 averages). The three acquisitions were acquired with different echo shifts to allow separate fat and water images to be generated. Finally, a semiautomatic image analysis was performed to segment and calculate the volumes of peritoneal and subcutaneous adipose tissues, respectively. Volumes of adipose depots are expressed in mL. Total adiposity is the sum of all peritoneal and subcutaneous depots and is expressed as a percent of body weight. Percent lean mass is calculated as (body weight in g)-0.9*(total adipose in mL)/(body weight in g).

### Histopathology, Immunostaining and Plasma Assays

Blood and liver tissue were collected after a 5-h fast. Plasma alanine aminotransferase (ALT) activity was measured using a commercial kit (Sekisui Diagnostics, LLC, Framingham, New York). Plasma triglyceride and cholesterol was measured with commercial kit on an Abbott Architect c2000 machine (Abbott, Abbott Park, Illinois). Plasma free fatty acids were measured with commercial kit (Biovision, Mountain View, CA). Liver tissue was fixed in 10% Formalin and embedded in paraffin. Fresh liver tissue was frozen in optimal cutting temperature (OCT) compound. Hematoxylin and eosin liver specimens were evaluated by light microscopy. Liver triglyceride determinations were measured biochemically using the Triglyceride Reagent Kit from Pointe Scientific Inc (Canton, MI) This procedure uses the glycerol phosphate oxidase (GPO) method based on the enzymatic determination of glycerol, generated by the enzymatic hydrolysis of triglyceride by the enzyme GPO after hydrolysis to hydrogen peroxide by lipoprotein lipase [30], [31]. Steatosis, inflammation, and ballooning were assessed in the livers of mice on the high fat and control diets by an experienced pathologist (Bettina G. Papouchado) in a blinded fashion. Steatosis, inflammation, and ballooning were scored based on NAFLD activity score (NAS) [32].

### Real-time PCR

Total RNA was isolated from liver tissue using RNeasy Tissue Mini kit (Qiagen, Valencia, CA). The reverse transcript was synthesized from 1 µg of total RNA using RETROCRIPT cDNA synthesis kit (Life Technologies, Carlsbad, CA). Real-time PCR quantification was performed. 25 µl of reaction mix contained: cDNA, Brilliant II Syber Green buffer (Agilent, Santa Clara, CA) and primers at final concentrations of 200 µM. RT-PCR was performed in the Mx3000P cycler (Agilent, Santa Clara, CA): 95 °C for 10 min, 40 cycles of 15 s at 95 °C, 30 s at 60 °C, 30 s at 72 °C followed by 1 min at 95 °C, 30 s at 55 °C and 30 s at 95 °C. The relative amount of target mRNA was determined using the comparative threshold (Ct) method by normalizing target mRNA Ct values to those of 18S, using MxPro 4.10 software (Agilent, Santa Clara, CA). Primers sequences are shown in Table 1.

**Table 1:** Primer sequences.

NCBI symbol	Gene	Forward 5'	Reverse 5'
Rn18s	18S	ACGGAAGGGCACCACCAGGA	CACCACCACCCACGGAATCG
Acc1	Acc1	ATGGGCGGAATGGTCTCTTTC	TGGGGACCTTGTCTTCATCAT
Acox1	Aox	TCCAGACTTCCAACATGAGGA	CTGGGCGTAGGTGCCAATTA
Apob	Apob	GCTCAACTCAGGTTACCGTGA	AGGGTGTACTGGCAAGTTTGG
Col1a1	Col1α1	ATGTTCAGCTTTGTGGACCTC	CAGAAAGCACAGCACTCGC
Cpt1a	CPT1α	TGGCATCATCACTGGTGTGTT	GTCTAGGGTCCGATTGATCTTTG
Fasn	Fas	AGCGGCCATTTCCATTGCCC	CCATGCCCAGAGGGTGGTTG
Il1	IL-1	CCAGGATGAGGACCCAAG	TCCCGACCATTGCTGTTT
Acadl	Lcad	GCTGCCCTCCTCCCGATGTT	ATGTTTCTCTGCGATGTTGATG
Fabp1	L-fabp	GTGGTCCGCAATGAGTTCAC	GTATTGGTGATTGTGTCTCC
Acadm	Mcad	AGGGTTTAGTTTTGAGTTGACGG	CCCCGCTTTTGTCATATTCCG
Ccl2	Mcp-1	AGGTCCCTGTCATGCTTCTG	TCTGGACCCATTCCTTCTTG
Mttp	Mttp	GTATTGGTGATTGTGTCTCC	TCTCTGTTGACCCGCATTTTC
Ppargc1b	Pgc1β	TCCTGTAAAAGCCCGGAGTAT	GCTCTGGTAGGGGCAGTGA
Ppara	Pparα	AGAGCCCCATCTGTCCTCTC	ACTGGTAGTCTGCAAAACCAAA
Pparg	Pparγ	TCGCTGATGCACTGCCTATG	GAGAGGTCCACAGAGCTGATT
Scd1	Scd1	CCGGAGACCCTTAGATCGA	TAGCCTGTAAAAGATTTCTGCAAACC
Acta2	αSma	GTCCCAGACATCAGGGAGTAA	TCGGATACTTCAGCGTCAGGA
Srebf1	Srebp1c	AACGTCACTTCCAGCTAGAC	CCACTAAGGTGCCTACAGAGC
Tnf	Tnfα	ATGAGCACAGAAAGCATGATC	TACAGGCTTGTCACTCGAATT

### Statistical Analysis

Values reported are means ± SEM. Data were analyzed by ANOVA, using the general linear models procedure (SAS, Carey, IN). The Shapiro-Wilk test was used to evaluate normality. Data were log transformed if needed to obtain a normal distribution. Least square means testing was performed for follow-up multiple comparisons. Mann-Whitney-U test was performed for analysis of histopathological NAFLD activity score between wild-type mice on the high fat and *Casp1* ^-/-^ mice on the high fat diet. Differences were considered to be statistically significant at *p*<0.05.

## Results

### High fat diet increases the expression of caspase-1 and IL-1β in the liver

**Figure 1:**
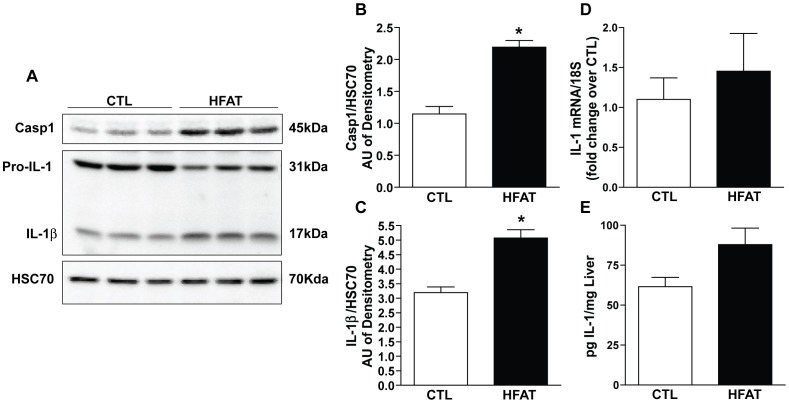
Caspase-1 and IL-1β expression is increased after high fat feeding. Livers of wild-type mice on the control or high fat diet were analyzed for pro-caspase-1 and IL-1β expression by immunoblot (A). Densitometric analysis was done for each protein normalized to HSC70 (B and C). mRNA for IL-1 expression in the liver of mice on the control or high fat diet was analyzed by RT-PCR (D). IL-1 was assessed by ELISA in whole liver tissue (E). Values with different superscripts are significantly different from one another (*p*<0.05). n = 4 control, n = 6 high fat.

C57BL/6 mice allowed free access to a high fat diet had increased hepatic expression of immunoreactive caspase-1 compared to mice on the control diet (Fig. 1a, b). Further, western blot analysis revealed that high fat diets also increased the quantity of mature IL-1β in the liver compared to mice of the control diet (Fig. 1a, c). Wild-type mice on the high fat diet also had increased expression of IL-1 mRNA expression in the liver; this increase was associated with increased total IL-1 protein in the liver, although not significantly(Fig. 1d, e.)

### Caspase-1 knockout mice develop obesity and increased adiposity on a high fat diet

**Figure 2:**
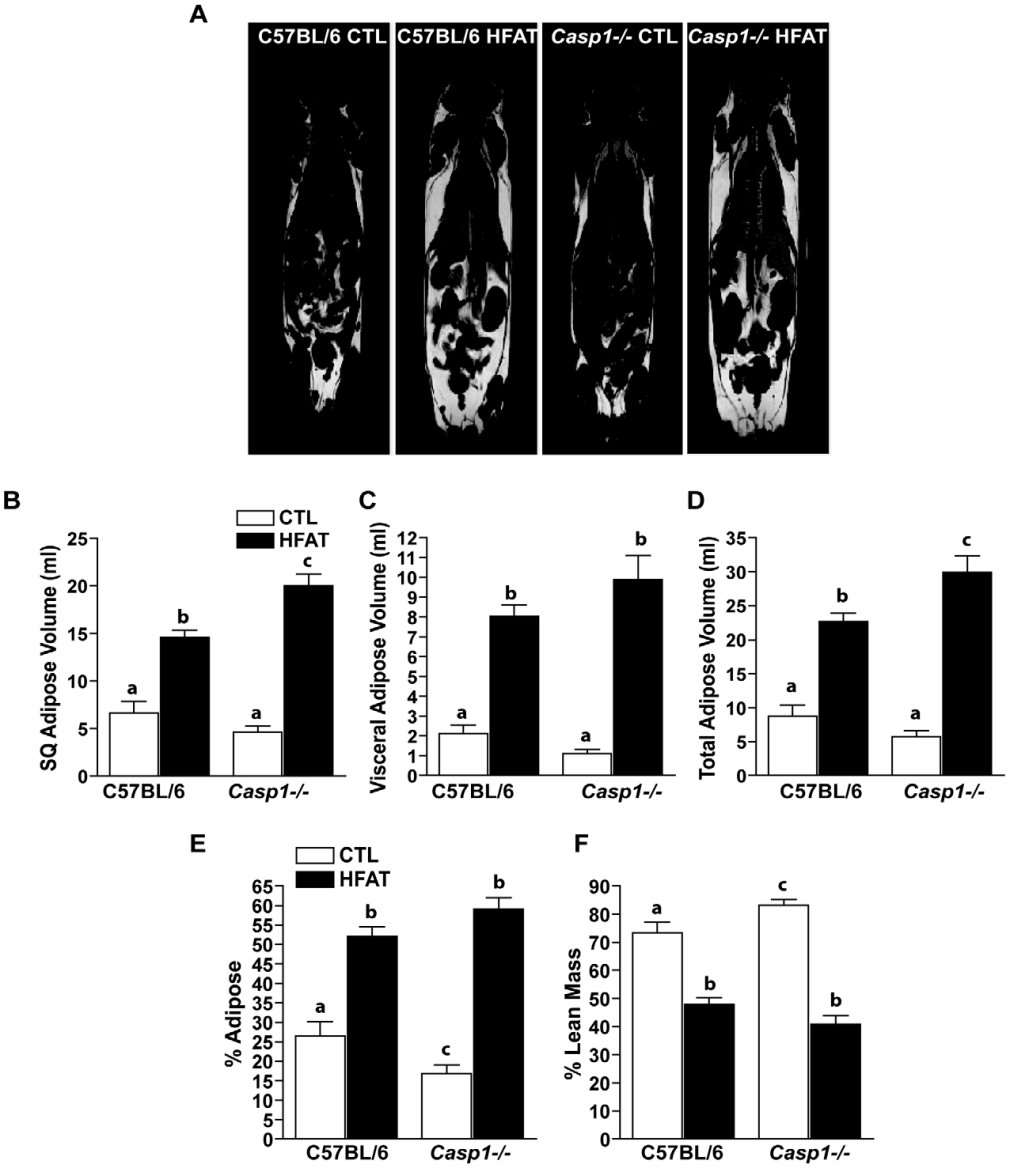
Adiposity is increased in caspase-1 knockout mice. Mice on the control and high fat diets were analyzed for body adiposity. Fat content of subcutaneous (B) and visceral (C) depots were calculated from three images. Total adipose volume was calculated from subcutaneous and visceral depots (D). Adiposity was normalized to body weight and expressed as% adipose (E). Lean mass was calculated based on the density of adipose tissue and normalized to body weight and expressed as% lean mass (F). Values represent means ± SEM. Values with different superscripts are significantly different from one another (*p*<0.05). n = 5 C57BL/6, n = 4 *Casp1^-/-^*.

If caspase-1 contributes to high fat induced liver injury, then caspase-1 knockout mice (*Casp1* ^-/-^) on a high fat diet should be protected from high fat diet-induced liver injury. Wild-type and *Casp1^-/-^* mice on high fat diets consumed similar amount of food and gained more body weight than control -fed mice, but *Casp1^-/-^* mice diet gained less than wild-type mice on the high fat diet (Table 2). Wild-type and *Casp1^-/-^* mice on both control and high fat diets had similar liver weight (Table 2). The adiposity of wild-type mice on the high fat diet was increased over mice on the control diet, measured by small animal magnetic resonance imaging (Fig. 2a). Wild-type and *Casp1^-/-^* mice on the high fat diet increased subcutaneous (Fig. 2b), visceral (Fig. 2c) and total body adipose tissue volume (Fig.2d) compared to mice on the control diet. Subcutaneous and total body adipose volumes were increased in *Casp1^-/-^* mice on the high fat diet to a greater extent than wild-type mice on the high fat diet (Fig. 2b and d). Both genotypes increased their percent adiposity/per g body weight and decreased their percent lean body mass after high fat diet feeding, but this shift was not affected by genotype (Fig.2e and f).

**Table 2:** Absence of caspase-1 protects from high fat-induced obesity.

	C57BL/6 Control	C57BL/6 High fat	*Casp1^-/-^* Control	*Casp1^-/-^* High fat
Initial Body Weight (g)	24.9±0.77	25.6±0.91	25.6 ±1.7	27.5±0.75
Final Body Weight (g)	29.3±1.0 *_a_*	39.4±0.91 *_b_*	32.6±1.5 *_b_*	39.4±1.6 *_b_*
Body Weight Change (g)	4.5±1.0 *_a_*	13.8±1.0 *_b_*	7.0±0.79 *_a_*	11.8±2.0 *_a_*
Food Intake (g)/Body Weight (g)/Week	0.42±0.071	0.31±0.014	0.42±0.018	0.34±0.015
Liver Weight (g)/Body Weight (g)	0.5±0.00	0.5±0.00	0.4±0.00	0.5±0.00

Data are represented as Mean ± SEM.

Wild-type (C57BL/6) and *Casp1^-/-^* mice were maintained on either a control or high fat diet for 12 weeks. Body weight at week 0 and 12 were measured. Food intake was measured weekly. Liver weight was measured at 12 weeks. Values represent means ± SEM. Values with different subscripts are significantly different from one another (*p*<0.05).

### The absence of caspase-1 prevents high fat diet-induced hepatic steatosis

**Figure 3:**
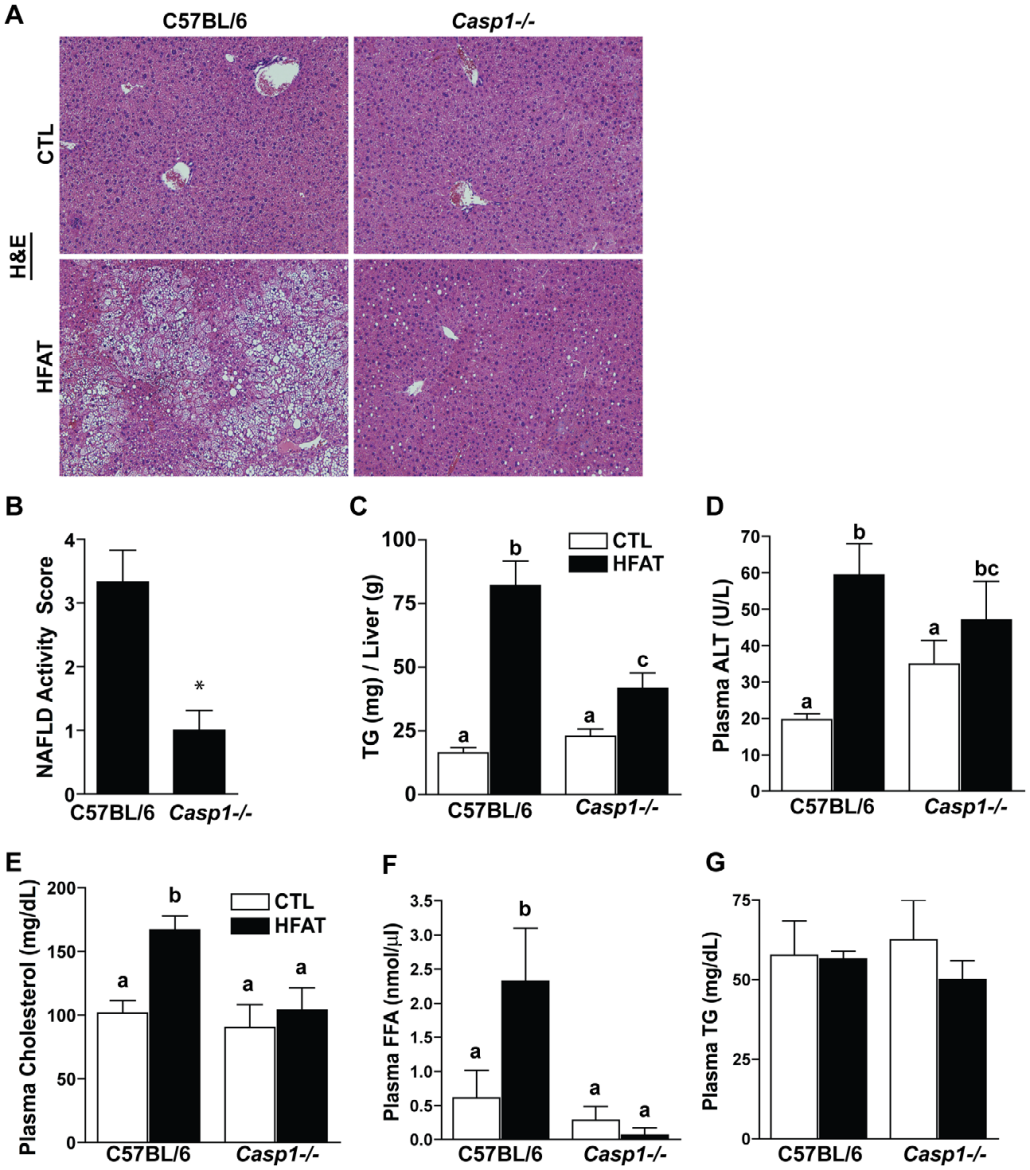
Caspase-1 knockout mice are protected from high fat-induced hepatic steatosis. Representative images of H&E stained livers in wild-type and *Casp1^-/-^* mice on control or high fat diet (10x) (A). NAFLD activity score of wild-type and *Casp1^-/-^* mice on the high fat diet was assessed by histopathology of H&E stained livers in a blinded-fashion by BPG (B). Hepatic triglyceride (TG) levels were measured biochemically from mice on the control or high fat diets (C). Plasma alanine aminotransferase (ALT) levels were analyzed from mice on the control or high fat diets (D). Plasma cholesterol (E), FFA (F) and TG levels (G) were measured biochemically as described in the methods section. Values represent means ± SEM. Values with different superscripts are significantly different from one another (*p*<0.05). n = 4 control, n = 6 high fat.

Wild- type mice fed a high fat diet had increased hepatic steatosis as assessed by H&E staining (Fig. 3a, Table 3). This response was reduced in the absence of caspase-1 (Fig. 3a). Histopathological analysis revealed the NAFLD Activity Score was greater in wild-type mice on the high fat diet than *Casp1^-/-^* mice on the high fat diet (Fig. 3b). Biochemical analysis revealed an increase in hepatic triglyceride wild-type mice fed high fat diet compared to control. *Casp1^-/-^* mice on high fat diet also had increased hepatic triglyceride compared to control, but was reduced compared to wild-type mice on high fat diet (Fig. 3c). Activity of plasma alanine aminotransferase (ALT), an indicator of hepatocyte injury, was elevated with high fat feeding; this increase was independent of genotype (Fig. 3d). Plasma cholesterol and free fatty acids (Fig. 3e, f) were increased in wild-type mice on high fat diet, but were attenuated in *Casp1^-/-^* mice on high fat diet. In contrast, plasma triglycerides were not affected by diet or genotype under the conditions of our investigation (Fig. 3g).

**Table 3:** Histopathological analysis of mice on the high fat diet.

	C57BL/6 Control	C57BL/6 High fat	*Casp1^-/-^* Control	*Casp1^-/-^* High fat
Steatosis	0±0	2.3±0.3	0±0	1.0±0.3
Ballooning	0±0	0.5±0.2	0±0	0±0
Inflammation	0±0	0.5±0.2	0±0	0±0

Pathologist: BPG. Data are represented as Mean ± SEM.

H&E slides of the livers of mice on the control and high fat diets were analyzed for steatosis, inflammation, and ballooning. H&E sections were scored in a blinded fashion by BPG. Values represent means ± SEM. n = 4 control, n = 6 high fat.

**Figure 4:**
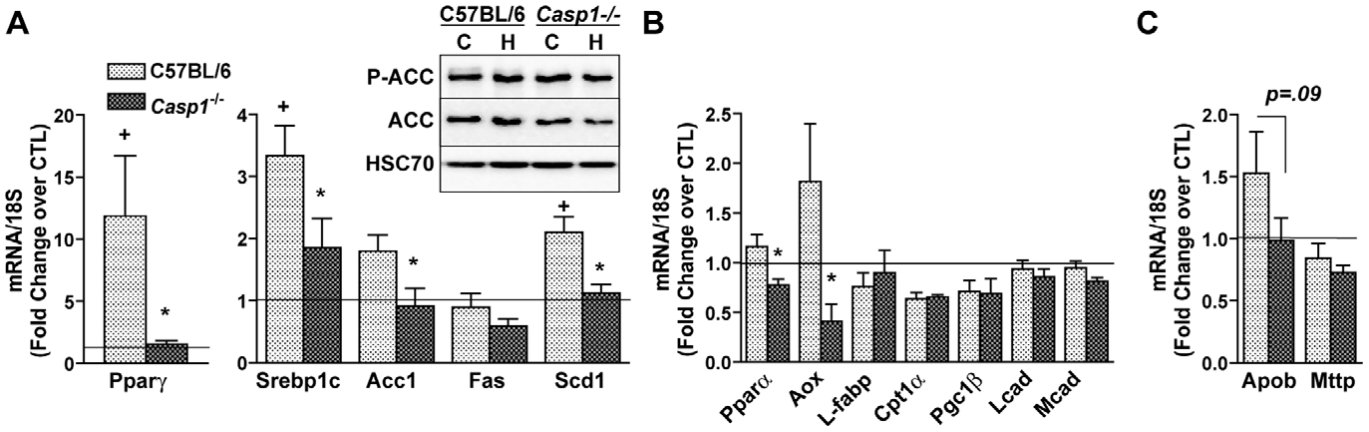
High fat-induced expression of lipogenesis-related genes is attenuated in caspase-1 knockout mice. Expression of mRNA for Pparγ, Srebp1c, Acc1, Fas and Scd1 were analyzed in the livers of wild-type and *Casp1^-/-^* mice on high fat diets and normalized to 18S. Hepatic expression of phospho-ACC and total ACC were analyzed by western blot (A). Expression of Pparα, Aox, L-fabp, Cpt1α, Pgc1β, Lcad and Mcad were analyzed in the livers of wild-type and Casp1-/- mice on high fat diet and normalized to 18S (B). Apob and Mttp mRNA were measured (C) in the liver of wild-type compared to and Casp1-/- mice on control or high fat diets and normalized to 18S (C). Values represent means ± SEM. Values with + different superscripts are significantly different from wild-type mice on control diet one another (p<0.05). Values with * are significantly different from wild-type mice on high fat diet (p<0.05). n = 4 control, n = 6 high fat.

To begin to investigate the mechanisms for lower hepatic steatosis in the *Casp1^-/-^* mice, expression of mRNA for genes associated with fatty acid synthesis, fatty acid oxidation and lipoprotein metabolism were measured by RT-PCR. Expression of mRNA for peroxisome proliferator-activated receptor gamma (Pparγ) and sterol receptor element binding protein-1c (Srebp1c), two key transcription factors regulating lipid synthesis, were increased with high fat feeding in wild-type mice; this induction by high-fat feeding was reduced in *Casp1^-/-^* mice (Fig. 4a). Consistent with the increase in srebp1c expression, stearoyl-CoA desaturase–1 (Scd-1), a downstream target of SREBP1c, was increased in wild-type mice on a high fat diet (Fig. 4a); the absence of caspase-1 attenuated this induction compared to wild type mice (Fig. 4a). In contrast, while expression of mRNA for acetyl Co-A carboxylase-1 (Acc1) was not significantly increased in wild type mice on the high fat diet (Fig. 4a), expression was lower in caspase1-deficient mice. Phosphorylation of ACC, nor total ACC was not affected by diet or genotype as assessed by western blot (Fig. 4a).

Expression of mRNA for peroxisome proliferator-activated receptor alpha (Pparα), a transcription factor regulating genes involved in lipid oxidation, and alternative oxidase (Aox) were not affected by high fat feeding in wild type mice, but were lower in *Casp1^-/-^* compared to wild-type mice (Fig. 4b). Expression of mRNA of additional genes related to fatty acid oxidation pathways (L-fabp, Cpt1α, Pgc1β, Lcad and Mcad) were not affected by either diet or genotype under the conditions of our study (Fig. 4b).

If *Casp1^-/-^* mice on high fat diet were protected from hepatic steatosis due to increased VLDL secretion, we would expect to observe an increase in expression of genes associated with the formation and/or secretion of VLDL in caspase-1 deficient mice compared to wild type mice. However, under the conditions of the current investigation, neither the expression of Apob or Mttp was increased in *Casp1^-/-^* mice on high fat diet compared to wild-type mice on high fat diet (Fig. 4c).

### Caspase-1 knockout mice are protected from high fat diet-induced markers of inflammation

**Figure 5:**
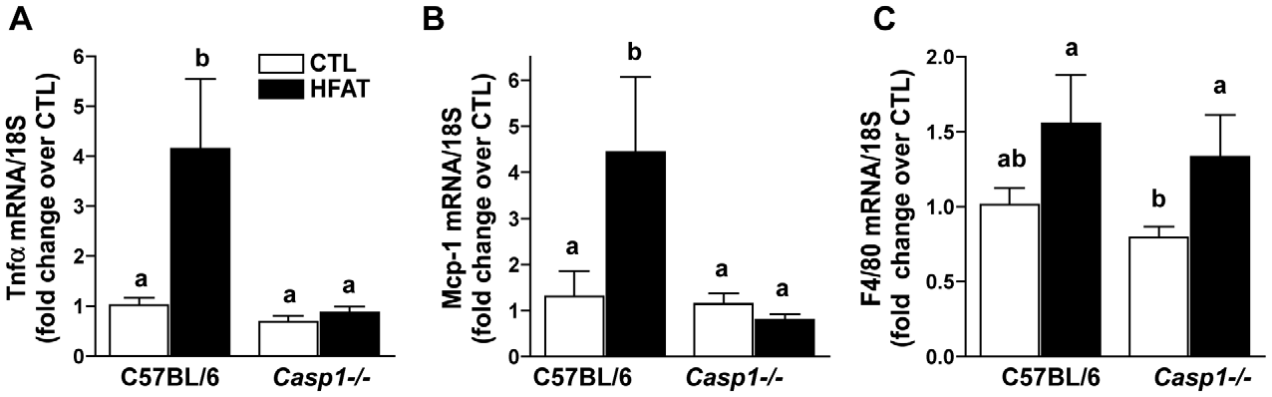
High fat-induced expression of inflammatory cytokines and chemokines is attenuated in caspase-1 knockout mice. Inflammatory cytokine/chemokine production in the liver is associated the early stages of NASH. mRNA expression for Tnfα (A), Mcp-1 (B) and F4/80 (C) in the liver of wild-type mice on control or high fat diets was performed by RT-PCR. Values represent means ± SEM. Values with different superscripts are significantly different from one another (*p*<0.05). n = 4 control, n = 6 high fat.

Since caspase-1 is a pro-inflammatory protease, its contribution to high fat –induced hepatic inflammation was investigated. Histopathological analysis of H&E stained liver sections displays inflammation in wild-type mice on the high fat diet compared to control diet (Table 3). Expression of mRNA for tumor necrosis factor alpha (Tnfα) and monocyte chemotactic protein-1 (Mcp-1) was increased with in wild-type mice on the high fat diet (Fig. 5a, b). Expression of these inflammatory markers was not increased in *Casp1^-/-^* mice on high fat diet (Fig. 5a,b). Expression of mRNA for pan-macrophage marker F4/80 was unchanged from control in both wild-type and *Casp1^-/-^* mice on the high fat diet (Fig. 5c).

### Early indicators of fibrogenesis are induced by high fat diet in wild-type, but not in Caspase-1 knockout mice

**Figure 6:**
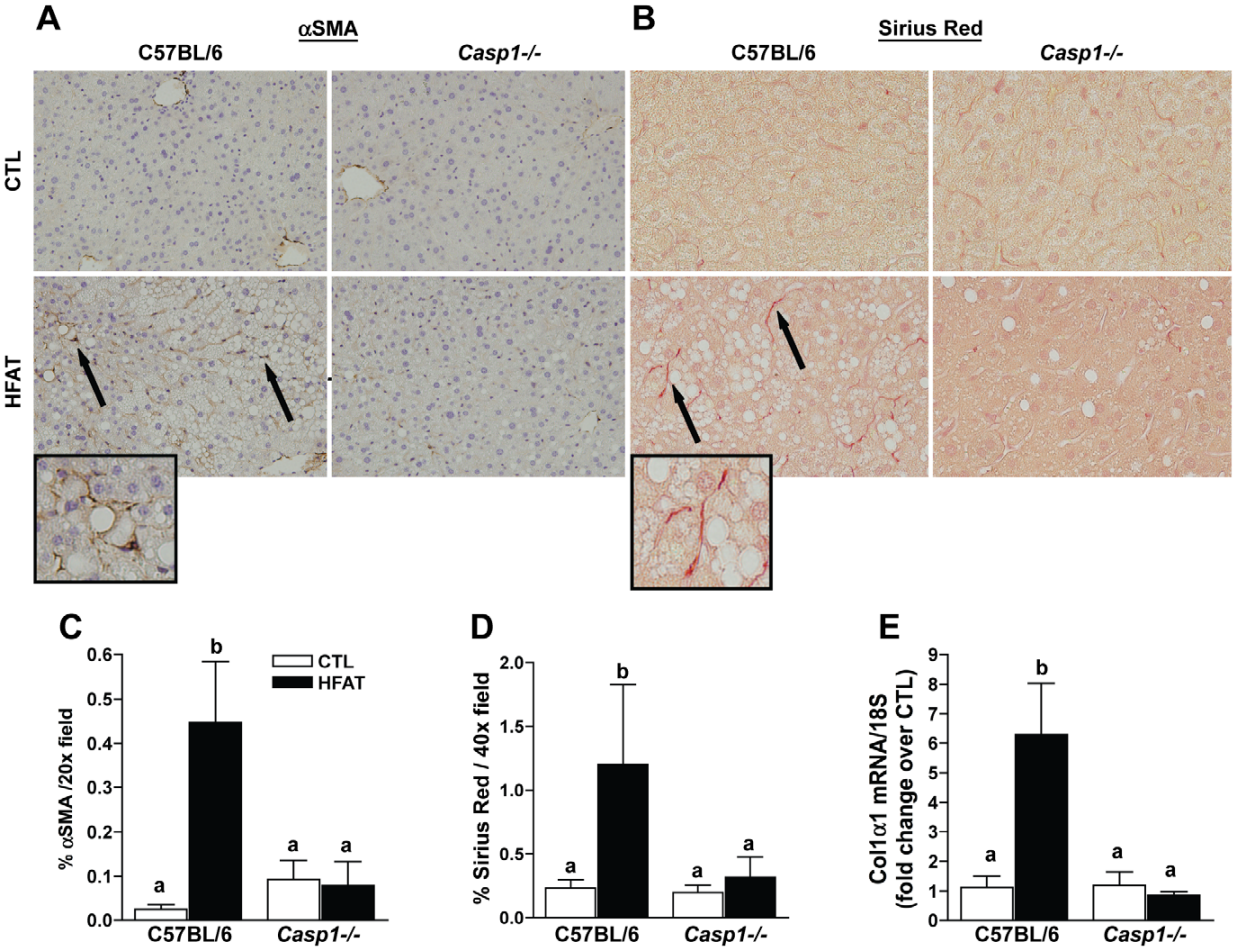
High fat-induced early fibrogenesis is prevented in caspase-1 knockout mice. Early to moderate fibrosis in the liver is associated with high fat diet feeding. αSMA immunohistochemistry (A, C), Sirius Red staining (B, D), Col1α1 mRNA (E) in the liver of mice on the control or high fat diets was performed by RT-PCR. Values represent means ± SEM. Values with different superscripts are significantly different from one another (*p*<0.05). n = 4 control, n = 6 high fat.

Given the recent findings of the inflammasome and caspase-1 in liver fibrosis [18], [21], [22], we evaluated the role of caspase-1 in high fat diet-induced early fibrogenesis. Expression of the hepatic stellate cell (HSC) activation marker alpha-smooth muscle actin (αSMA) by immunohistochemistry was increased in the perisinusoidal space of the livers of wild-type mice on the high fat diet compared to control, but not in *Casp1^-/-^* mice (Fig. 6a, c). Sirius Red staining, an indicator of extracellular matrix deposition, was greater in wild-type mice on the high fat diet compared to control diet and was present in a chicken-wire manner (Fig. 6b, d). However, in the absence of caspase-1, a high fat diet did not induce extracellular matrix deposition over mice on the control diet (Fig. 6b and d). Consistent with this finding, mRNA expression for collagen type 1-alpha-1 (COL1α1) was increased in the sinusoidal space in wild-type mice on a high fat diet; but was not increased *Casp1* ^-/-^ mice (Fig. 6e).

## Discussion

The progression of NAFLD and NASH involves the development of hepatic steatosis, increased expression of inflammatory cytokines and chemokines, and the eventual deposition of extracellular matrix proteins. This process involves complex interactions between multiple cell types within the hepatic architecture. The contribution of cells of the innate immune system and HSCs are particularly critical to the progression from NAFLD to NASH. The purpose of this study was to test the hypothesis that caspase-1 plays a role in the pathogenesis of high fat diet-induced NASH. Caspase-1 and IL-1β expression were increased in mice on the high fat diet. While both wild-type and *Casp1^-/-^* mice became obese on the high fat diet, *Casp1^-/-^* mice were protected from a number of the phenotypic characteristics of early NASH. For example, the high fat diet increased hepatic steatosis and expression of lipogenesis related genes in wild-type mice, but *Casp1^-/-^* mice were protected from these diet-induced phenotypes. These data indicate that caspase-1 directly and/or indirectly influences hepatocytes in the development of fatty liver. Further, while the high fat diet increased inflammatory cytokine and chemokine expression in wild-type mice, this response was attenuated in *Casp1^-/-^* mice, suggesting a role for caspase-1 in macrophages during the progression to NASH. Finally, our data also indicate that caspase-1 plays an important role at early stages of fibrogenesis in response to high fat diets. Evidence for activation of HSCs and accumulation of extracellular matrix proteins was apparent in wild-type mice after 12 weeks of high fat diet. Importantly, *Casp1^-/-^* mice had decreased fibrogenesis compared to wild-type in response to a high fat diet, suggesting either a direct and/or indirect role of caspase-1 in the activation of HSCs.

*Casp1^-/-^* mice fed a high fat diet exhibited reduced accumulation of liver triglycerides during high fat feeding, associated with greater adiposity than wild-type mice. Increased adiposity in *Casp1^-/-^* mice may contribute to their protection from diet-induced steatosis. Subcutaneous adipose, rather than visceral adipose, is increased in *Casp1^-/-^* mice on high fat diet. This may be protective, as subcutaneous adipose is considered metabolically benign in comparison to visceral adipose tissue, which is associated with insulin resistance and the metabolic syndrome. However, within the scope of this investigation, we cannot determine if the shift in adipose accretion to subcutaneous adipose tissue directly protects from diet-induced hepatic steatosis in caspase1-deficient mice or whether it is merely an association.

Reduced accumulation of hepatic triglycerides in the *Casp1^-/-^* mice might also be due to decreased hepatic lipogenesis, since the *Casp1^-/-^* mice expressed less mRNA for genes associated with hepatic lipogenesis, Pparγ, Srebp1c and its downstream targets Acc1 and Scd1. Further, protection from high-fat diet-induced steatosis in caspase1-deficient mice was not likely due to increased fatty acid oxidation, as expression of mRNA for Pparα and Aox was also lower in *Casp1^-/-^* mice on high fat diet compared to wild-type mice, indicative of a reduced capacity for lipid oxidation.

Another way in which caspase-1 may contribute to steatosis is via regulation of cytokine and chemokine expression in the liver. TNFα promotes the maturation of SREBP-1 [33] and MCP-1 may directly regulate hepatic lipid homeostasis, independent of its chemokine activity [34]. Thus, lower expression of hepatic TNFα and MCP-1 in *Casp1^-/-^* mice compared to wild-type mice on the high fat diet likely contributed to a decrease in high fat diet-induced hepatic steatosis.

The expression of caspase-1 and other components of the inflammasome is constitutive in monocytes. Activation of the inflammasome in monocytes is therefore primarily controlled by recruitment of proteins to the inflammasome complex. In contrast, expression of inflammasome components and caspase-1 can be induced in non-myeloid cells, such as hepatocytes or other non-parenchymal cells [35]. Thus, the inflammasome and caspase-1 is differentially regulated in multiple cell types of the liver; activity in each cell type may make unique contributions to hepatic pathophysiology during disease progression. Caspase-1 has been localized to both hepatocytes and non-parenchymal cells of the liver after MCD feeding and contributes to inflammation and fibrogenesis in this model of NASH [18]. However, very little further data is available to explain the cell-type specific mechanisms responsible for promoting caspase-1 activity and inflammasome formation in NASH.

The role of LPS activation of TLR-4 on hepatic Kupffer cells, leading to expression of pro-inflammatory cytokines and chemokines, during high fat diet induced liver injury is well studied. Importantly, TLR4-/- mice are protected from high fat diet induced liver injury [36]. TLR ligands can include both pathogen-associated molecular patterns (PAMPs) (eg. lipopolysaccharide (LPS)) and damage-associated molecular patterns (DAMPs) (eg. free fatty acids (FFA). High fat diet feeding not only increases the availability of FFA, but also increases plasma LPS to a concentration equivalent to that seen in low-level metabolic endotoxemia and bacterial overgrowth [37]. This metabolic endotoxemia is considered a key inducer of metabolic syndrome [37], with multiple cytokines (Tnfα and IL-1β) and chemokines (e.g. Mcp-1) contributing to the progression of both alcoholic-liver disease (ALD) and NAFLD [34], [38], [39], [40].

Ganz et. al. recently reported that LPS, a ligand for TLR4, may be an important regulator of hepatic expression of caspase-1 and the NLRP3 inflammasome [21]. These data provide an important potential link between the inflammasome and TLR-4-dependent responses. Our data are consistent with the hypothesis that caspase-1 contributes to inflammatory cytokine and chemokine expression in the liver during high fat diet feeding. When wild-type mice were fed the high fat diet for 12 weeks, expression of hepatic Tnf-α and Mcp-1 mRNA were increased; this response was attenuated in *Casp^-/-^* mice. This difference might be due to differential recruitment of monocytes/macrophages or differences in macrophage activation between wild-type and caspase-deficient mice. Here we find that high fat diet-induced increases in expression of monocyte/macrophage marker F4/80 was not affected by genotype, suggesting that caspase-1 is important for regulating the sensitivity of Kupffer cells to activation during high fat diet-induced obesity, rather than recruitment and/or proliferation of Kupffer cells in the liver.

In addition to a role in steatosis and inflammatory cytokine activity, caspase-1 also contributed to early stages of hepatic fibrosis in high fat diet-induced obesity. Fibrogenesis in the liver requires the activation of hepatic stellate cells, characterized by an increase in expression of αSMA expression. αSMA -positive cells were increased in the livers of wild-type mice on the high fat diet, but were reduced in the parenchyma of *Casp1^-/-^* mice. Wild-type mice on the high fat diet also increased expression of Col1α1 and a deposition of chicken-wire-like extracellular matrix in the perisinusoidal space, indicative of early phases of fibrogenesis. *Casp1^-/-^* mice were protected from these markers of early fibrogenesis in response to a high fat diet. These data suggest that caspase-1 is important to the activation of HSC and promotes high fat diet–induced early fibrogenesis.

These data add to the increasing evidence for an innate immune function of HSCs. For example, TLRs are expressed by HSC [41] and have been associated with the progression of fibrosis. *TLR4^-/-^* mice are protected from bile duct ligation, thioacetamide or carbon tetrachloride –induced fibrosis [42]. Further, inflammasome components are expressed by both LX-2 cells and primary cultures of HSC, regulating HSC function in culture and in vivo during experimental liver fibrosis induced by carbon tetrachloride or thioacetamide [15]. Mice lacking inflammasome components ASC and NLRP3 have reduced thioacetamide or carbon tetrachloride -induced liver fibrosis [15]. However, the cellular and molecular mechanisms for caspase-1 -dependent HSC activation and fibrogenesis remain to be elucidated and will require further investigation.

Taken together, the present data suggest that caspase-1 is expressed and functional in multiple cell types of the liver at different times of high fat diet-induced NASH, promoting steatosis, inflammatory cytokine production and the activation of HSC leading to early fibrogenesis. This data is consistent with the concept that the inflammasome is differentially regulated in monocytic and non-monocytic cells. Caspase-1 plays a role in protecting non-monocytic cells, such as hepatocytes, from steatosis [17], whereas it contributes to the production of inflammatory cytokines in monocytic cells, like Kupffer cells. Data from both high fat diet (presented here) and MCD diet-induced [18] liver injury clearly indicates that caspase-1 also contributes to the regulation of HSC function in different mouse models of NASH, indicating a need for further studies investigating the regulation and role of the caspase-1 inflammasome in hepatic stellate cells.
